# Emerging adults not in education, employment or training (NEET): socio-demographic characteristics, mental health and reasons for being NEET

**DOI:** 10.1186/s12889-018-6103-4

**Published:** 2018-10-25

**Authors:** Raúl A. Gutiérrez-García, Corina Benjet, Guilherme Borges, Enrique Méndez Ríos, María Elena Medina-Mora

**Affiliations:** 1 Epidemiologic and Psychosocial Research, National Institute of Psychiatry Ramón de la Fuente Muñiz, Mexico City, Mexico; 2 Psychological Research, De La Salle Bajio University, Salamanca, Guanajuato, Mexico

**Keywords:** Youth, Employment, Education, Mental health, Mexico

## Abstract

**Background:**

A growing group of emerging adults in many countries around the globe are not incorporated into the education system or the labor market; these have received the label “NEET: not in education, employment nor training”. We describe the mental health and socio-demographic characteristics of emerging adults who are NEET from Mexico City (differentiating between NEET who are homemakers and NEET who are not) compared to their peers who are studying, working or both, in a city in which education and employment opportunities for youth are limited. A secondary objective, because of the often inconsistent inclusion criteria or definitions of NEET, was to evaluate the heterogeneity amongst NEET emerging adults in terms of their perceived reasons for being NEET and to evaluate whether different reasons for being NEET are associated with different mental health characteristics.

**Methods:**

The participants were 1071 emerging adults aged 19 to 26; they were interviewed in person by an interviewer in their homes as part of a follow-up study of the Mexican Adolescent Mental Health Survey. The Composite International Diagnostic Interview (WMH-CIDI) assessed psychiatric disorders, substance use and abuse, suicidal behavior and socio-demographic characteristics.

**Results:**

Of the total sample, 15.3% were NEET homemakers, 8.6% NEET non-homemakers, 41.6% worked only, 20.9% studied only and 13.5% worked and studied. Of those who were NEET, 12.6% were NEET by choice. NEET non-homemakers had overall greater odds of substance use, substance use disorders and some suicidal behaviors in comparison with all their peers, whereas NEET homemakers had reduced odds. Those who were NEET because they didn’t know what to do with their life had greater odds of mood, behavioral, and substance disorders, use of all substances and of suicide behaviors compared to those who were NEET by choice.

**Conclusions:**

Non-homemaker NEET who lack life goals require targeted mental health intervention. The demographic reality of emerging adults not in education or employment and the varying reasons they give for being NEET are not consistent with how NEET is often conceptualized in terms of a societal problem.

## Background

Emerging adulthood is a recent concept proposed by Arnett in 2000 [1–3], and adopted by many American [4, 5] and European [6–9] researchers, to encompass a stage of life occurring roughly between the ages of 18 and 26. The conceptual development of this new life stage is a response to changes in industrialized countries, such as later ages of adult roles like marriage, parenthood and work, an increase in the years dedicated to education and professional qualification, and thus a prolonged period of exploration of possible life directions [10]. These aspects of emerging adulthood contribute to this being one of the most demographically heterogeneous stages of life (in terms of employment, studying, marital status, having children, living or not with one’s family of origin), with no distinct normative reference [11]. This concept, however, is conceptualized primarily in terms of psychological development, whereas Bynner [12] argues that this stage of life is more greatly influenced by structural and social factors such as employment and educational opportunities.

How well this concept of emerging adulthood represents the experience of youth in developing countries and differing cultural contexts is starting to be investigated. Initial findings suggest that emerging adulthood, as described for Western developed countries, is not the norm in Latin America, particularly in lower socioeconomic levels [13–15]. The five psychological characteristics proposed by Arnett to describe emerging adults in developed countries include instability, possibilities, self-focus, in-betweenness, and identity exploration. While individuals in this age group in Latin America report some of these characteristics, younger ages of first marriage or union and of first parenthood, cultural attitudes towards living with one’s family of origin until first marriage or even after, coupled with more limited educational and employment opportunities, certainly makes emerging adulthood in this context distinct. In Mexico, the context of economic crisis hinders the access of emerging adults to key social institutions for their development, such as education and work; this limited access contributes to a process of social exclusion, instability and vulnerability in this population [16], which can cause adulthood postponement and low autonomy [17].

Research suggests college entrance and entry into the labor market typically takes place during emerging adulthood, and that the successful transition from school to work is a societal expectation for this stage [18]. Reality, however, deviates from social expectations as there is a significant proportion of the population of emerging adults who do not follow this path, partly due to limitations in access to higher education and high unemployment; this leads potentially to youth growing up faster even though they lack traditional employment opportunities. A growing group of emerging adults is not incorporated into the education system or the labor market; these have received the label “NEET: not in education, employment nor training” [19–21]. The National Institute of Statistics and Geography (INEGI) in Mexico defines NEET as all those above 14 years of age that are unemployed (whether or not they are actively looking for work and whether or not they are available to work with the exception of the severely disabled) and do not attend school [22].

However, this one-size-fits-all label masks the varied situations of these emerging adults not in education or employment. A person may be in this situation because of 1) inability to find employment, 2) inability to gain entrance into college or other levels of schooling 3) lack of economic resources to continue studying, 4) informal employment options, 5) lack of social recognition of unpaid work, 6) suffering from an illness, 7) taking time off to explore possibilities or because one is undecided about life plans, or 8) alternative life paths [23]. Work done outside formal employment structures (such as care work) is as important as work done within formal employment structures (market work) and should be recognized as such; however young adults, primarily females who are homemakers, are sometimes classified as NEET, for example the OECD [24] includes as NEET those who participate in the functions of caring for people and being housewives.

Mexico has had an economic crisis in recent years which has promoted the growth of informal work; 64% of youth do not have access to formal employment (in other words, employment that is taxed, monitored and subject to labor laws) leading to informal work (that which is off the books, tends to be precarious and can be exploitative) [25]. The National Institute of Statistics and Geography (INEGI) in Mexico reported that most informal work is carried out mainly as domestic work in other people’s homes and in agriculture [26]. In population studies, certain characteristics are over-represented among NEET youth. The key findings to date tend to be demographic and social factors, specifically, low socioeconomic status [27, 28], parental factors (eg, low educational attainment, divorce, parental unemployment), living arrangements (eg, not living with either parent, homelessness), and negative school experiences (eg, low educational attainment, bullying, persistent truancy, expulsion and suspension, behavioral problems, learning difficulties) [29]. In most cases, very little attention is paid to mental health factors.

Both emerging adulthood and adolescence involve many life transitions and significant mental health risks. Fifty percent of people who develop a mental disorder do so before the age of 21 [30]. In Mexico, prior evidence from the Mexican Adolescent Mental Health Survey suggests that NEET adolescents aged 12 to 17 had greater psychopathology, substance use and suicidal behavior when compared to teens who studied exclusively [31], and subsequently had poorer mental health compared to their peers as they transitioned to early adulthood [32].

The conditions that lead to NEET status, as well as the experience of NEET status, may impact upon mental health through social disengagement and marginalization [33, 34]. However, the causes and consequences of being NEET are likely to be different in emerging adulthood (a stage of greater socio-demographic heterogeneity) than in adolescence when NEET is a more deviant situation because by law adolescents should be in school [35]. In Mexico, compulsory education was the conclusion of middle school at age 15 until the year 2012, at which time compulsory education was extended to include high school.

Prior studies have shown that mental disorders are associated with lower educational attainment and higher risk of unemployment [36]. One such study found that 19% of youth seeking primary care attention in Australia were not engaged in study or work [37]; these youth were mostly male, had a criminal record, risky cannabis use, greater depressive symptomatology, more advanced mental illness, and poor social skills. In another study NEET youth in Britain had higher rates of mental health problems and substance abuse than non-NEET peers [38].

Therefore, the objective of this study was to describe the mental health and socio-demographic characteristics of emerging adults not in education or employment, termed NEET (differentiating between NEET who are homemakers and NEET who are not) compared to their peers who are studying, working or both, in a city in which education and employment opportunities for youth are limited. A secondary objective, because of the often-inconsistent inclusion criteria or definitions of NEET, was to evaluate the heterogeneity amongst NEET emerging adults in terms of their perceived reasons for being NEET and to evaluate whether different reasons for being NEET is associated with different mental health characteristics.

## Methods

### Participants

The participants were 1071 emerging adults aged 19 to 26; they were interviewed in person by an interviewer in their homes in 2013 as part of a follow-up study of the Mexican Adolescent Mental Health Survey, a general population representative survey of adolescents in the Mexico City Metropolitan Area conducted in 2005 [39]. Five groups were defined: 1. NEET who are homemakers, 2. NEET who are not homemakers, 3. those who study and work, 4. those who work only, and 5. those who study only; then, they were compared independently. We separated the NEET into homemakers and non-homemakers, as homemakers are included in some definitions of NEET (such as in the Mexican governmental definition), but not others, and we wanted to explore how they may be similar or different from each other and from their non-NEET peers. The NEET homemaker category included those who self-identified as homemakers. The NEET non-homemaker category included those who receive no financial compensation for work, those looking for employment, and those who are not enrolled in any educational institution.

### Assessment

The World Mental Health version of the WHO Composite International Diagnostic Interview 3.0 (WMH-CIDI) [40], a fully structured diagnostic interview, assessed psychiatric disorders using DSM-IV criteria [41], suicidal behavior, substance use, employment, education and other socio-demographic factors. The WMH-CIDI included the assessment of the following disorders in the 12 months prior to the interview: mood disorders (major depressive disorder, bipolar I and II, and dysthymia), anxiety disorders (specific phobia, social phobia, separation anxiety disorder and generalized anxiety disorder), substance use disorders (alcohol, tobacco and drug abuse and dependence), and behavioral disorders (attention-deficit hyperactivity disorder, oppositional-defiant disorder, conduct disorder and intermittent explosive disorder). A section on substance use asked about tobacco use, alcohol and drugs (marijuana, cocaine, tranquilizers or stimulants used without a medical prescription, heroin, inhalants, and other drugs) in the previous 12 months. A section on suicidality asked about suicidal ideation, plans and attempt in the previous 12 months. Participants were asked why they were in the situation of not working or studying and their answers were categorized as: to perform household duties, not finding work or gaining school admission, by choice, not knowing what to do with their life and other reasons that did not fit the aforementioned categories.

### Procedures

Emerging adults were recruited from the contact information that they gave as part of their prior participation in the Mexican Adolescent Mental Health Survey (MAMHS). Of the original sample, 91.9% gave contact information to be re-contacted. Of those, 89.4% were located eight years later. A response rate of 62.0% of located and eligible participants was obtained (participants were eligible if they continued to live in Mexico City and were not in prison or a hospital), though this was only 35.6% of the MAMHS sample. To make sure that the participants that were re-interviewed did not vary from those that were not re-interviewed in ways that might affect our results, we tested for possible attrition bias by performing χ2 tests, to evaluate possible differences in baseline socio-demographic and mental health characteristics of those participants that participated in this current survey versus those that did not. We found no differences in lifetime DSM-IV disorders between MAMHS respondents that participated in the current survey and those that did not. The variables that showed bias (i.e., sex, being a student, and living with both parents) were used to calculate weights to ensure that the current participants represented the initial MAMHS sample [42].

The Internal Review Board of the National Institute of Psychiatry approved the study. Fieldwork was carried out by survey research firm and supervised by the research team at the National Institute of Psychiatry. Therefore, we carried out extensive training and in situ supervision of field interviewers. These field interviewers located selected participants in their homes and after explaining the study, asked for their informed consent.

### Analyses

We weighted the data to adjust for differential probabilities of non-response and post-stratified by age and sex to represent the age and sex distribution of this age group in the population and to be representative of the wave I sample. We tabulated the weighted proportions and standard errors using the SUDAAN 11.0.1 statistics software for the five education/employment groups and then by reason for being NEET. To estimate the association of psychiatric disorder, substance use and suicidal behavior with education/employment status group and reason for being NEET we computed multivariate logistic regressions, and from the average marginal predictions from these fitted models we calculated adjusted odds ratios (aOR). Tables 1, 2 and 3, each present the results of a single multivariate multinomial logistic regression model; in each model all mental health variables (psychiatric disorders, substance use, suicidal behavior) are entered as independent variables, socio-demographic variables (sex, married, has children, some college education, living with family of origin) as covariates and education/employment category as the dependent variable with three levels such that the mental health characteristics of NEET homemakers and NEET non-homemakers are compared to the reference group (those who work only on Table 1, those who study only on Table 2 and those who work and study on Table 3) controlling for their sociodemographic characteristics. Table 4 presents the results of a multivariate logistic regression model in which all mental health variables are entered as independent variables, socio-demographic variables as covariates and type of NEET as the dependent variable with NEET non-homemakers as the reference group. Finally, Table 5 presents the results of single multivariate multinomial logistic regression model among the NEET youth only, in which all mental health variables are entered as independent variables, socio-demographic variables as covariates and reason for being NEET as the dependent variable with 4 levels, the reference group being those who are NEET by choice.

**Table 1:**
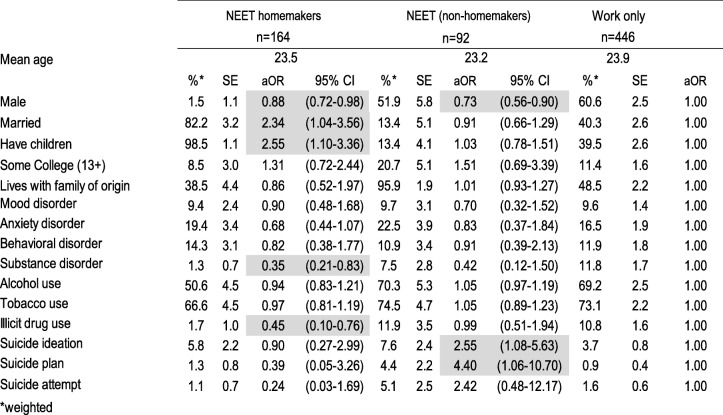
Socio-demographic characteristics, psychiatric disorders, substance use and suicidal behavior of NEET versus working emerging adults, Mexico City 2013

*weighted

Shading shows statistically significant associations at *p* < 0.05

**Table 2:**
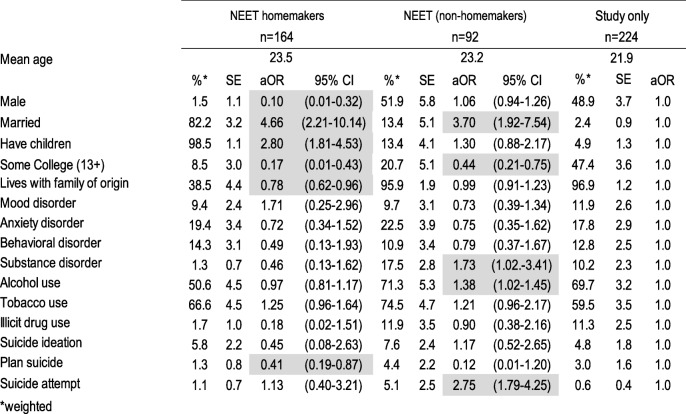
Socio-demographic characteristics, psychiatric disorders, substance use and suicidal behavior of NEET versus student emerging adults, Mexico City 2013

*weighted

Shading shows statistically significant associations at p < 0.05

**Table 3:**
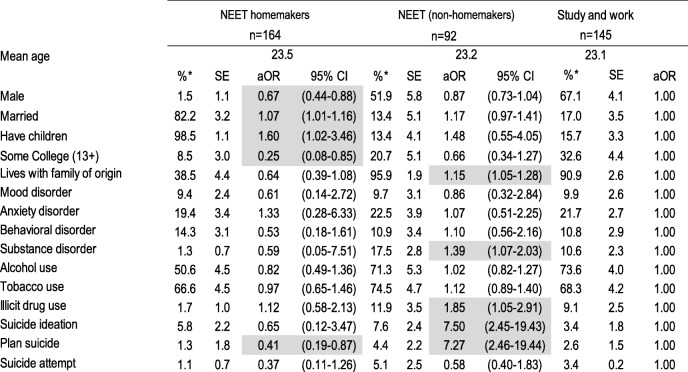
Socio-demographic characteristics, psychiatric disorders, substance use and suicidal behavior of NEET versus studying and working emerging adults, Mexico City 2013

*weighted

Shading shows statistically significant associations at p < 0.05

**Table 4:**
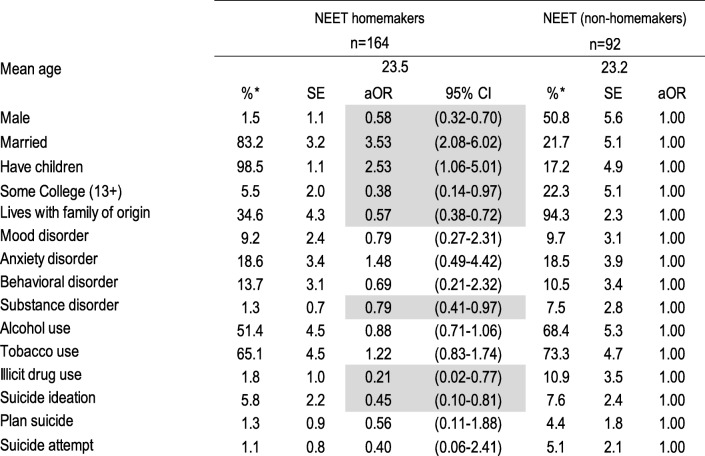
Socio-demographic characteristics, psychiatric disorders, substance use and suicidal behavior of homemaker versus non-homemaker NEET, Mexico City 2013

*weighted

Shading shows statistically significant associations at p < 0.05

**Table 5:**
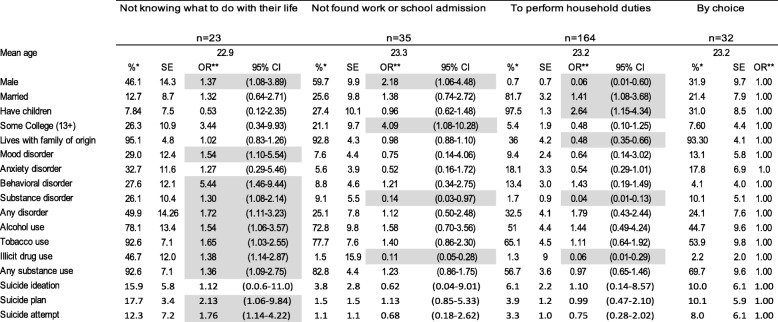
Socio-demographic characteristics psychiatric disorders, substance use and suicidal behavior of emerging adults by reasons for being NEET, Mexico City 2013

**adjusted for age and sex

*Note*: Early adult outcomes (dependent variables) were placed in rows, and reasons NEET (the independent variable) in the columns for ease of visual display

Shading shows statiscally significant associations at *p* < 0.05

## Results

Of the total sample, 15.3% were NEET homemakers, 8.6% NEET non-homemakers, 41.6% worked only, 20.9% studied only and 13.5% worked and studied. Table 1 shows the socio-demographic characteristics, psychiatric disorders, substance use and suicidal behavior of NEET versus working emerging adults. NEET homemakers were mostly female (98.5%), married (82.2%), had children (98.5%), roughly a third lived with their family of origin (38.5%), and few had attained any college education (8.5%). Only 6.3% of NEET homemakers were NEET by choice. Of NEETs who were not homemakers, roughly half were male (50.8%), few were married (21.7%), most lived with their family of origin (94.3%), and less than a quarter had attained any college education (22.3%). Almost 19 % of them were NEET by choice. When compared to those who worked exclusively, NEET homemakers were less likely to be male, to have a substance use disorder, and use illicit drugs (aORs ranging from 0.35 to 0.88) whereas they were more likely to married and to have children (aOR = 2.34; 95% CI = 1.04–3.56 and aOR = 2.55; 95% CI = 1.10–3.36, respectively). On the other hand, NEET who were not homemakers, compared to those who worked exclusively, were more than twice as likely to have suicide ideation (aOR = 2.55 95%CI = 1.08–5.63) and more than four times more likely to plan suicide (aOR = 4.40 95%CI = 1.06–10.70); they were also less likely to be male (aOR = 0.73; 95%CI = 0.56–0.90).

Table 2 presents socio-demographic characteristics, psychiatric disorders, substance use and suicidal behavior of NEETs versus students. NEET homemakers, compared to those who studied exclusively, were more likely to married and to have children (aOR = 4.66; 95% CI = 2.21–10.14 and aOR = 2.80; 95% CI = 1.81–4.53, respectively), but less likely to be male, to have any college education, to live with their family of origin and to plan suicide (aORs ranging from 0.10–0.78). NEET who were not homeworkers, compared to those who studied exclusively, were less likely to have any college education (aOR = 0.44; 95% CI = 0.21–0.75), but more likely to be married, to have a substance use disorder, to use alcohol, and to have made a suicide attempt (aORs ranging from 1.38 to 2.75).

Table 3 shows socio-demographic characteristics, psychiatric disorders, substance use and suicidal behavior of NEET versus studying and working emerging adults. NEET homemakers compared to those who studied and worked, are more likely to be married and to have children (aOR = 1.07; 95% CI = 1.01–1.16 and aOR = 1.60; 95% CI = 1.02–3.46, respectively), but were less likely to be male, to have any college education, and to have made a suicide plan (aORs ranging from 0.25 to 0.67). NEET who were not homeworkers, compared to those who studied and worked, were more likely to live with their family of origin, to have a substance use disorder, illicit drug use, suicide ideation and a suicide plan (ORs ranging from 1.15 to 7.50).

Socio-demographic characteristics, psychiatric disorders, substance use and suicidal behavior of NEET that are homemakers versus non-homemakers are shown on Table 4. NEET homemakers, compared to those who are non-homemakers, were less likely to be male, to have any college education, to live with their family of origin, to have a substance use disorder, illicit drug use and suicide ideation (aORs ranging from 0.21 to 0.79).

Table 5 presents the socio-demographic characteristics, psychiatric disorders, substance use and suicidal behavior of emerging adults by reasons for being NEET. The most reported reason for being NEET was to perform household duties (64.5%). The next most frequently reported reasons for being NEET were not finding work or not being admitted to any school (13.8%), being NEET by choice (12.6%), and not knowing to do with their life (9.1%). Two participants reported other reasons. We, therefore, considered them as missing data for the analyses. Those who were NEET because of not knowing what to do with their life, in comparison to those who were NEET by choice, were more likely to be male, to have a mood disorder, a behavioral disorder, a substance disorder, alcohol use, tobacco use, illicit drug use, a suicide plan and a suicide attempt (aORs ranging from 1.30 to 5.44).

NEET because of not finding work or gaining school admission, compared to those NEET by choice, were more likely to be male and have some college education (ORs = 2.18 and 4.09, respectively), but were less likely to have a substance use disorder and illicit drug use (ORs = 0.14 and 0.11). NEET to perform household duties, compared to those who are NEET by choice, were more likely to have children and to be married (ORs = 2.64 and 1.41), and less likely to be male, to live with their family of origin, to have a substance use disorder, and illicit drug use (ORs ranging from 0.06–0.48).

## Discussion

Almost a quarter of the interviewed emerging adults from Mexico City were NEET, an estimation consistent with the 27% reported for the general population of youth aged 14–29 years living in Mexico [43]. However, we found that not all NEET were equally vulnerable to mental health conditions. A large proportion of these NEET were homemakers and NEET homemakers overall had less substance use, substance use disorders and some suicidal behaviors in comparison with all of their age-group peers, whereas NEET non-homemakers had greater substance use, substance use disorders and suicidal behavior compared to all their age-group peers. In fact, the most at risk emerging adults were non-homemaker NEET who didn’t know what to do with their life, a group that would be included in most definitions of NEET. Our data suggest that NEET in emerging adulthood may be experienced differently depending on the reason for being NEET and is not the same phenomena as NEET in adolescence. This is likely due to the heterogeneity of NEET emerging adults and that divergent life paths at this stage are more normative and less deviant than at earlier stages of life. For example, being a NEET homemaker is socially acceptable at this stage of life in the Mexico City context and thus does not present a mental health challenge.

The primary reason for being NEET, given by 62% was domestic duties. Those who gave this reason were almost exclusively female (99%) and married (81.7%). For this group, the transition to adult roles has mainly been made and it is unlikely they experience emerging adulthood as posited by Arnett [1] for developed countries. While Arias and Hernández [17] found that Mexicans aged 16 to 34 (particularly those in their twenties) largely endorsed agreement with descriptions of their life stage similar to those posited in the theoretical framework developed by Arnett for emerging adults, their study of Mexicans included primarily college students, and much fewer working persons and those with children than would be expected in a representative population study, and thus, likely represents the perspective of Mexicans from a higher socioeconomic and educational level, and not this group of NEET dedicated to domestic activities.

The second most important reason given for being NEET, (endorsed by 13.7% of all NEET and almost a fifth of non-homemaker NEET), reported they were NEET because they were unable to find employment or gain school entrance. This group most closely represents what is considered the primary reason for NEET among many academics and policy makers, lack of educational and employment opportunities for youth in a difficult economic climate. Unexpectedly, this group had less risk of a substance use disorder and illicit drug use than those NEET by choice. The lack of vulnerability found in this group, may perhaps be due to this being a temporary situation not long enough to impact upon their functioning.

In a similar proportion, NEET by choice was endorsed by 12.6% of all NEET (almost a fifth of non-homemaker NEET). While still primarily females (69%), those saying they were NEET by choice were less likely to be married and have children and almost exclusively lived with their family of origin. This group may be experiencing emerging adulthood more closely to the way it is characterized for developed countries, particularly in terms of identity exploration and postponement of adult roles.

A smaller proportion, (9%) reported being NEET because they did not know what to do with their lives. Forty-six percent male, this group is also likely in the process of identity exploration. This is the group with the greatest psychopathology. They had an increased risk of a mood disorder, a behavioral disorder, a substance use disorder, each type of substance use, suicidal plan and a suicide attempt. Suicidal behavior may reflect a negative view of the future in those that feel lost or lack life purpose. For example, Kleinman and Beaver found that meaning in life and search for meaning in life were associated with decreased suicidal ideation over time and reduced lifetime odds of a suicide attempt [44]. They also found that meaning in life partially mediated the association of perceived burdensomeness and thwarted belongingness (two factors that may be present in some NEET) with suicidal behavior. Furthermore, social exclusion has been found to be related to decreased meaning in life [45]. All this may explain the increased risk of suicidal behavior and emotional upheaval in those NEET who are in this situation due to lack of life direction. Additionally, substance use and mood disorders may contribute to disengagement, either because of the impairment caused through symptoms, such as apathy, reduced motivation and goal-directedness, and difficulties to make decisions. On the other hand, these problems may follow as a consequence of reduced social interactions and a lot of unstructured time leaving them lonely and without a sense of purpose. In a study of Mexican and Spanish NEET many reported loneliness [46]. Fergusson, McLeod and Horwood found that longer durations of unemployment were associated with increases in depression, alcohol and illicit substance abuse/dependency and other adverse psychosocial outcomes accounting for between four and 14 % of the risk. Less support in that longitudinal study was found for reverse causal explanations of prior psychosocial burden predicting unemployment. Directionality and causality, however, cannot be determined in this current study [47].

The varying reasons emerging adults in Mexico gave for being NEET and the most frequent reason (household responsibilities), however, do not conform to the general concept of NEET generally espoused by policy makers or the media when referring to NEET. For example, they generally don’t consider homemakers as part of the concept of NEET [48], nor those actively looking for employment. In Europe policy makers have been concerned that NEET youth may opt out of civic participation having lost trust in institutions and thus may be at risk of radicalization [49], whereas the Mexican media consider NEET youth to be vulnerable prey for organized crime [50, 51]. Thus, the demographic reality of emerging adults not in education or employment, and the varying reasons they give for being NEET, are not consistent with how NEET is often conceptualized in terms of a societal problem.

### Strengths and limitations of this study

A limitation of this study is that we did not consider the amount of time or duration the individuals have been NEET, which might be associated to mental health or reasons for being NEET. Studies of unemployment have shown an association between ill mental health and duration of unemployment [52, 53]. There may be other reasons for being NEET which we did not address. Additionally, causal direction cannot be determined given the cross-sectional data.

Despite these limitations, a strength of this study is shedding light upon emerging adulthood in a country culturally, socially and economically different from where the majority of studies on emerging adults have been conducted and on a group of emerging adults considered to be vulnerable especially in contexts of limited educational and employment opportunities. An important contribution of this study is providing a more layered interpretation of the figures that agencies typically report for the number of NEET by understanding the various reasons for being NEET. We included all emerging adults that are not engaged in education, employment or training in our categorizations of NEET, and did not exclude any due to a preconceived notion of NEET (such as those engaged in domestic activities or searching for employment). Therefore, we were able to provide a greater understanding of this phenomenon in the context of heterogeneous life trajectories in emerging adulthood.

## Conclusions

In conclusion, these results have important public policy implications. Strategies to facilitate the transition from school to work and those particularly focused on disengaged young adults need to consider the varied reasons for their disengagement, with focused attention on the mental health needs of those who are NEET because they don’t know what to do with their lives. Conversely, NEET homemakers have comparable or even better mental health than their same age peers and, rather than being considered a disadvantage, their unremunerated work should be recognized.

As we have shown, a considerable number of NEET emerging adults in our sample had clinically relevant levels of symptoms corresponding to established diagnostic criteria. In light of the age of the cohort studied, an age with low health service utilization due to generally good physical health, and low health coverage especially among the unemployed, targeted treatment and population-based interventions in non-health sector spheres is needed; these might include internet-based or mobile application interventions or interventions provided through community recreation centers. The absence of school and work in almost a quarter of these emerging adults is relevant for public health initiatives targeting individuals in this developmental stage, because psychiatric illness indirectly and directly poses significant risk to emerging adult health [54].

## Abbreviations

95% CI: 95% Confidence interval
aOR: Adjusted Odds Ratios
DSM-IV: Diagnostic and Statistical Manual of Mental Disorders, Fourth Edition
INEGI: National Institute of Statistics and Geography
MAMHS: Mexican Adolescent Mental Health Survey
NEET: Not in education, employment nor training
OECD: The Organisation for Economic Co-operation and Development
WMH-CIDI: The World Health Organization World Mental Health Composite International Diagnostic Interview
